# Ultrasound Diagnosis in Small Ruminants: Occurrence and Description of Genital Pathologies

**DOI:** 10.3390/vetsci9110599

**Published:** 2022-10-28

**Authors:** Mário Felipe Alvarez Balaro, Isabel Oliveira Cosentino, Ana Clara Sarzedas Ribeiro, Felipe Zandonadi Brandão

**Affiliations:** Faculdade de Veterinária, Universidade Federal Fluminense, Rua Vital Brazil, 64, Vital Brazil, Niteroi 24230-340, Rio de Janeiro, Brazil

**Keywords:** reproductive disorders, sheep, goat, ultrasonographic diagnosis

## Abstract

**Simple Summary:**

Reproductive pathologies represent a significant barrier to achieving a highly productive flock, which makes securing a fast, low-invasiveness, and low-cost diagnosis very important. In this research, we report on an eight-year retrospective study (2012–2020) of ultrasound data carried out in Rio de Janeiro State, Brazil, to explore the occurrence and appearance of certain reproductive tract pathologies of small ruminants. A total of 3463 animals from 16 sheep flocks (1688 ewes and 55 rams) and ten dairy goat flocks (1704 does and 16 bucks) raised under extensive and intensive management systems, respectively, were used in the study. All animals were submitted to ultrasound examination of their reproductive tracts, which occurred during the breeding and outbreeding seasons. From the 8.98% of does that presented reproductive disorders, the most frequent finding was hydrometra (50.98% of cases), while for 4.14% of ewes presenting disorders, recent fetal loss (22.85%) and cystic endometrial hyperplasia (20%) were the most frequent. For males, 43.63% and 56.25% of rams and bucks, respectively, presented disorders; for rams mainly microlithiasis. Ultrasonography provided clinically useful information about the reproductive tract, both in terms of the disorders and their stages, making the establishment of a diagnosis easier, and also making it possible to improve therapy, as well as the prognosis prediction.

**Abstract:**

This study reports the occurrence and appearance of various reproductive tract pathologies of small ruminants diagnosed using ultrasound. An eight-year retrospective study of collected ultrasound data was carried out in Rio de Janeiro State, Brazil from September 2012 to July 2020. A total of 3463 animals from 16 sheep flocks and 10 dairy goat flocks, raised under extensive and intensive management systems, respectively, were used in the study. All animals were submitted to an ultrasound examination of their reproductive tract. Data were analyzed using Fisher’s exact test (*p* < 0.05), to compare disorder frequencies within and between species. Ewes (4.14%) had fewer reproductive disorders than does (8.98%), while rams (43.63%) and bucks (56.25%) presented no significant differences. Hydrometra was the most frequent finding in does, represented by 50.98% of cases; while, in ewes, recent fetal loss (22.85%) and cystic endometrial hyperplasia (20.00%) were the most frequent. In bucks, the findings showed no clear differences; while, for rams, the most frequent finding was testicular microlithiasis (75.00%). Ultrasonography offers clinically useful information about the reproductive tract via the images it provides; knowledge of which makes it possible to improve the diagnosis, therapy, and prognosis of genital pathologies.

## 1. Introduction

Sound knowledge of the reproductive tract’s main pathological affections can guide veterinary practitioners in the proper diagnosis and treatment of related diseases, as well as allow the adoption of suitable preventive and control measures, for optimal fertility, reproduction, and production [1,2]. Hence, the improvement of diagnosis methods for diseases that affect genital systems, such as congenital and acquired abnormalities—important causes of subfertility or infertility in these species [3,4,5]—may lead to better productivity.

Applying ultrasound to the genital tract of small ruminants is a diagnostic tool in reproductive management that has been widely adopted around the world [6,7,8]. It is an imaging modality that is non-invasive and practical, as well as offering real-time results [9]. In recent decades, obstetric ultrasound has been widely used in small ruminants, administered transcutaneously or transrectally, and with the aim of diagnosing pregnancy, quantifying fetus numbers, sexing, and fetal viability [7,10,11,12]. Moreover, the use of this tool in goats and sheep has also been highlighted for the diagnosis of pathologies in ovaries and non-pregnant uteri [2,13,14], as well as for the andrological evaluation of testicles and sex glands [8,14,15,16,17,18].

Some barriers for the practitioner are still present, however. Crilly et al. [9] pointed out that the absence of ultrasonographic descriptions of the common pathologies, associated with the reluctance of some farmers to conduct the examination of single animals, contributes to a deficiency in interpreting findings that differ from pregnancy diagnosis. Furthermore, studies related to the occurrence of pathologies in the reproductive tract of small ruminants are scarce, with those that are available being related to *post mortem* findings in slaughterhouses or clinical findings [19,20,21]. In this sense, the practitioner would benefit from the knowledge accrued via the prevalence of ultrasonographic description, in the context of the main diseases. In this manner, new information being made accessible could significantly improve the field use of ultrasonographic diagnosis and, consequently, also improve treatment and prognosis.

Thus, this study aims to report the occurrence of in vivo diagnosis of genital disorders in commercial goat and sheep flocks. Additionally, there is a focus on characterizing the B-mode and Doppler ultrasound of genital tract disorders.

## 2. Materials and Methods

This study was conducted with the full approval of the Ethics Committee of Universidade Federal Fluminense, under license number 814/2016. A retrospective study (from September 2012 to August 2020) was delineated from data of the Grupo de Estudo, Pesquisa e Extensão em Caprinos e Ovinos (GEPECO) from the Faculty of Veterinary, Universidade Federal Fluminense, Brazil. The data were obtained from ultrasound examinations for pregnancy diagnosis and andrological examinations performed in 11 sheep flocks (1688 ewes and 55 rams, Santa Inês, Dorper, and crossbred animals) as part of an extensive or semi-intensive system, and in 10 dairy goat flocks (1704 does and 16 bucks; mainly Saanen, but also Boer goats) of an intensive system, applied during the breeding and outbreeding seasons. All flocks were located in the states of Rio de Janeiro and Minas Gerais, Southeastern Brazil. 

All animals were submitted to ultrasound examination of the reproductive tract (Sonoscape S6, SonoScape, Yizhe Building, Yuquan Road, Shenzhen, China), using a 7.5-MHz linear transrectal transducer, both for transrectal (taped to a PVC tube) and transcutaneous (scrotal evaluation) analysis. Using a syringe, 10 mL of carboxymethylcellulose gel (Carbogel UTL; Carbogel Indústria e Comércio LTDA, São Paulo, Brazil) was also inserted into the animal’s rectum for lubrication and to increase contact between the rectum’s wall and the transducer. For the testis scan, a small area of the scrotum was shaved before the carboxymethylcellulose gel was used for contact enhancement and image formation. During the ultrasound, animals were restrained by an assistant to keep them in a standing position while an experienced examiner searched for any image indicating morphophysiological changes in the animals’ reproductive tracts. In case any disorders were detected, B-mode and color-Doppler mode (when necessary) videos of the genital tract were recorded, to inform subsequent evaluations. The Doppler settings used for the genital assessments consisted of 20% color gain, pulse repetition frequency (PRF) 1.0 kHz, 7-cm depth, and wall filter (WF) 75 MHz.

Varying diagnostic methodologies were utilized to confirm the ultrasound findings, depending on the disorder found. Unfavorable prognosis, tumor, or obstetric disorders were confirmed by surgery or slaughter and necropsy with histopathological evaluation (when necessary). Luteal and follicular cysts were assessed using videolaparoscopy. Other cases were diagnosed according to the response to the recommended treatment (e.g., hydrometra, endometritis) and prospective ultrasound follow-up, although the disorder was computed only once during the study.

Data were organized in spreadsheets (Excel 2019^®^, Microsoft Office, São Paulo, Brazil) and analyzed by performing Fisher’s exact test, to compare the ultrasonographic disorder frequencies both within and between species. A Chi-square test was performed to determine if any species presented significantly more disorders. Differences were considered significant when *p* ≤ 0.05.

## 3. Results

**Figure 1:**
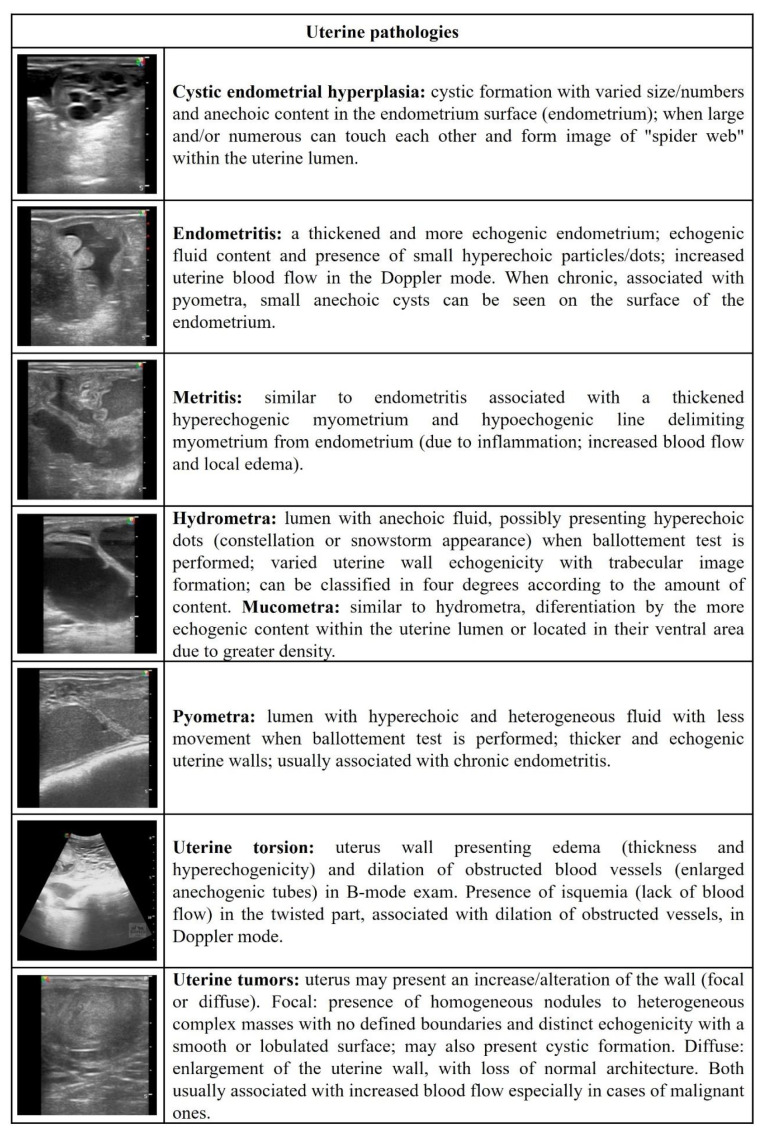
Ultrasonographic description of uterine pathologies in ewes and does.

**Figure 2:**
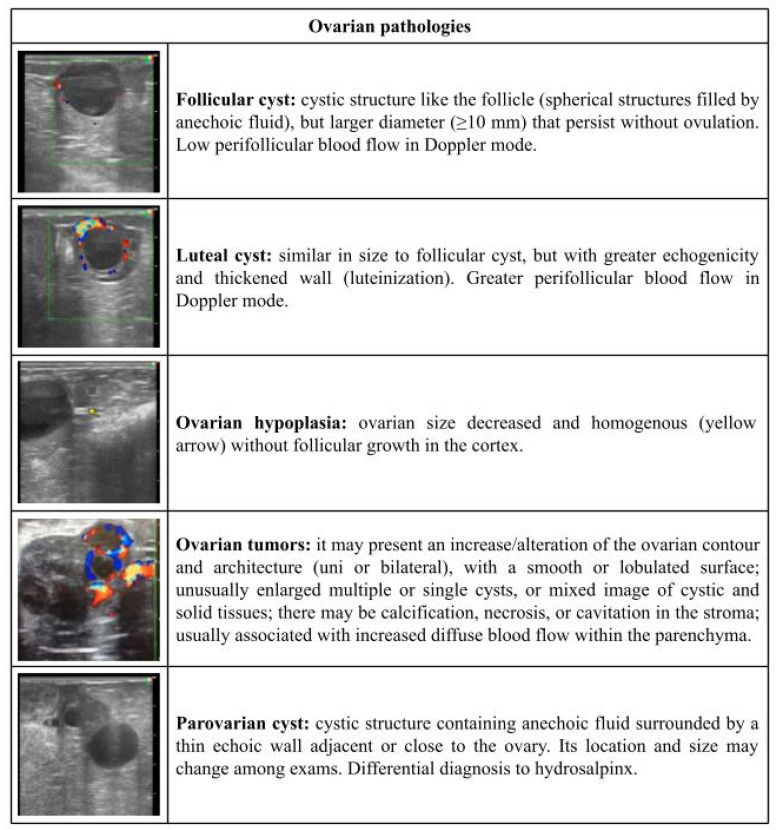
Ultrasonographic description of ovarian pathologies in ewes and does.

**Figure 3:**
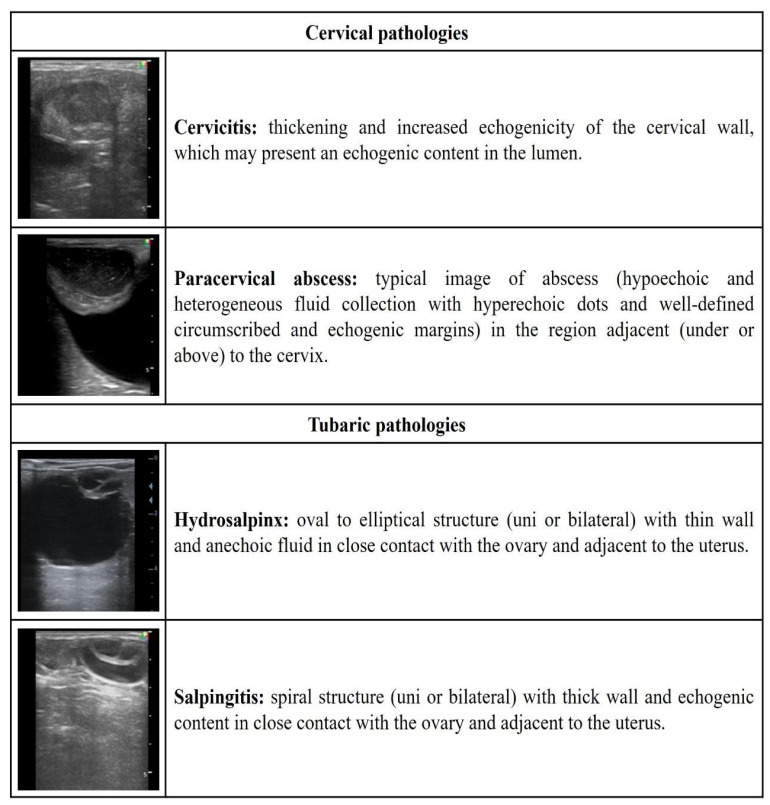
Ultrasonographic description of cervical and tubaric pathologies in ewes and does.

**Figure 4:**
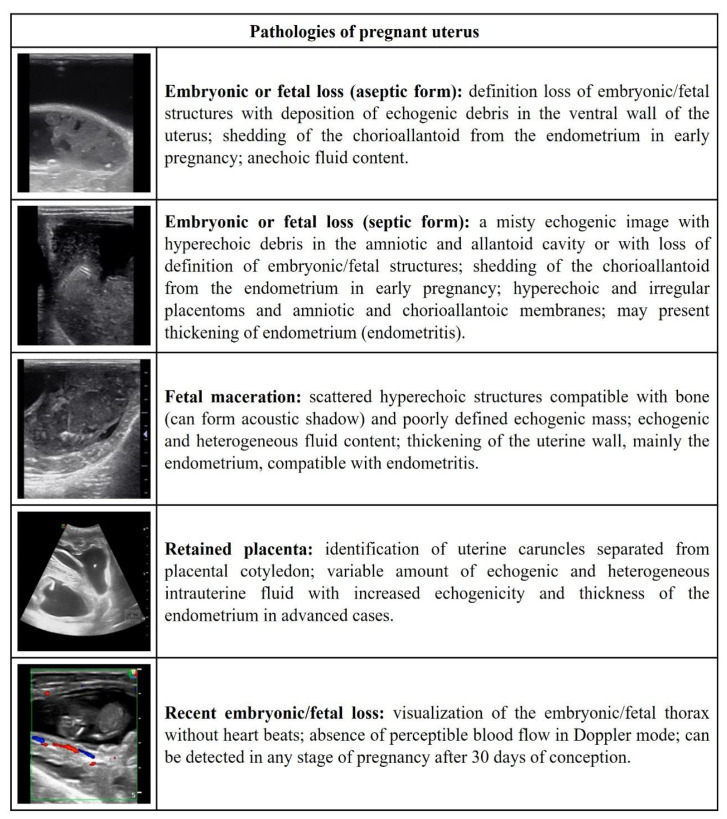
Ultrasonographic description of the pathologies of the pregnant uterus in ewes and does.

**Figure 5:**
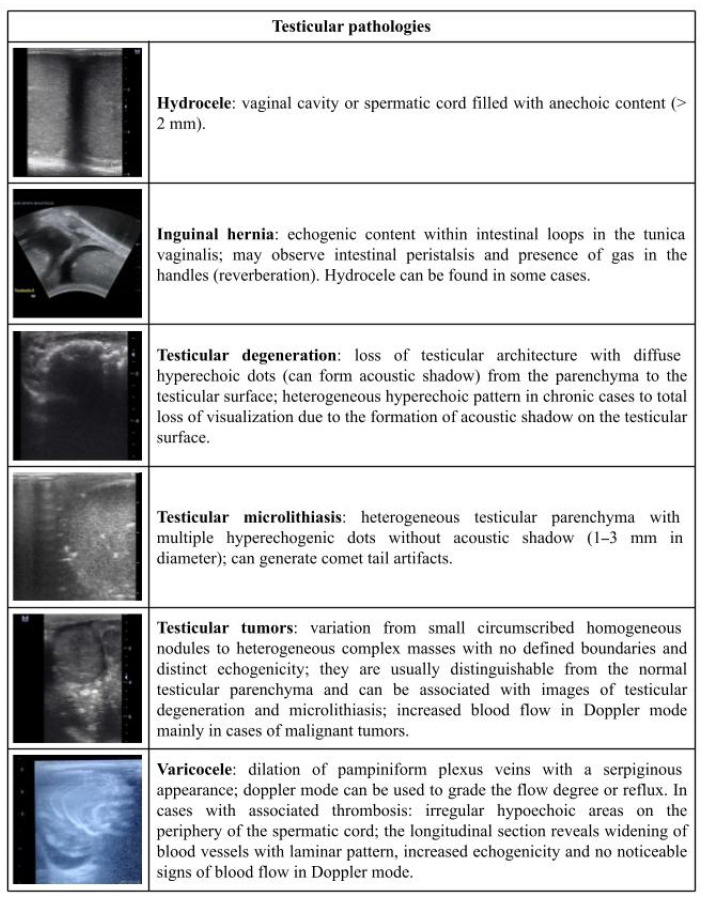
Ultrasonographic description of testicular pathologies in rams and bucks.

Figure 1, Figure 2, Figure 3, Figure 4 and Figure 5 describe the sonographic characterizations of the main genital disorders diagnosed in sheep and goats. Video recordings were also included as Supplementary Materials (Video S1, Video S2, Video S3, Video S4, Video S5, Video S6, Video S7, Video S8, Video S9, Video S10, Video S11, Video S12, Video S13, Video S14, Video S15, Video S16, Video S17, Video S18, Video S19, Video S20, Video S21, Video S22, Video S23, Video S24, Video S25 and Video S26). Ewes (4.14%; 70/1688) had fewer (*p* < 0.05) reproductive disorders than does (8.98%; 153/1704), while rams (43.63%; 24/55) and bucks (56.25%; 9/16) presented no difference (*p* > 0.05).

**Figure 6:**
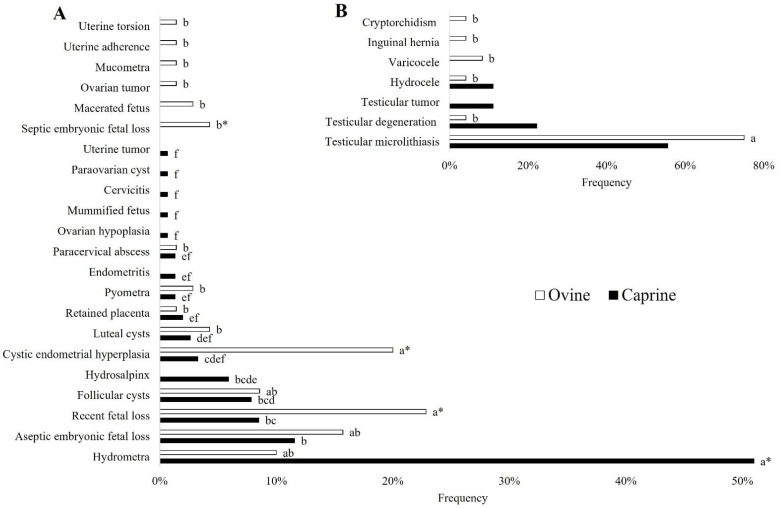
(**A**) Occurrence of female reproductive tract disorders in ewes and does. (**B**) Occurrence of male reproductive tract disorders in rams and bucks. ^a, b, c, d, e, f^ Different letters among disorders in the same species indicate differences (Fisher’s exact test; *p* < 0.05); * Means difference between species (Fisher’s Exact Test; *p* < 0.05).

Among the 153 cases of reproductive disorders found in does, hydrometra was the most frequent finding (*p* < 0.05), represented by 50.98% (78/153) of cases. Subsequently, the presence of aseptic embryonic fetal loss (11.76%; 18/153), recent fetal loss (8.49%; 13/153), follicular cysts (7.84%; 12/153), and hydrosalpinx (5.88%; 9/153) were the median findings. Finally, the occurrence of cystic endometrial hyperplasia (3.27%; 5/153), luteal cysts (2.61%; 4/153), retained placenta (1.96%; 3/153), pyometra, endometritis, and paracervical abscess (1.31%; 2/153), ovarian hypoplasia, mummified fetus, cervicitis, paraovarian cyst, and uterine tumor (0.65%; 1/153) was less frequent (Figure 6A).

From the 70 cases of reproductive disorders presented by ewes, recent fetal loss (22.85%; 16/70) and cystic endometrial hyperplasia (20.00%; 14/70) were the most frequent findings (*p* < 0.05). Other findings included the presence of aseptic embryonic fetal loss (15.71%; 11/70), hydrometra (10.00%; 7/70), and follicular cyst (8.57%; 6/70). Finally, the occurrence of a luteal cyst and septic embryonic fetal loss (4.28%; 3/70), pyometra and macerated fetus (2.85%; 2/70), ovarian tumor, placenta retention, mucometra, uterine adherence, uterine torsion, and paracervical abscess (1.42%; 1/70) was less frequent (Figure 6A).

In bucks, as shown in Figure 6B, no difference was identified among the findings: testicular microlithiasis (55.55%; 5/9), testicular degeneration (22.22%; 2/9), and testicular tumor and hydrocele (11.11%; 1/9). In rams, testicular microlithiasis presented as the most frequent finding (*p* < 0.05), occurring in 75.00% (18/24) of the 24 cases found. In comparison, varicocele (8.33%; 2/24), inguinal hernia, testicular degeneration, hydrocele, and cryptorchidism (4.16%; 1/24) presented as the least frequent findings (Figure 6B).

## 4. Discussion

Ultrasonography of the reproductive tract provides a sensitive and practical tool for detecting the diversity of congenital or acquired genital disorders in small ruminants. To the best of the authors’ knowledge, this is the first study in which such a methodology has been applied for determining occurrences of the most frequent genital pathologies and for comparing findings between sheep and goats. 

Ultrasonographic characterizations of the main reproductive disorders in small ruminants can enhance diagnosis, as well as contribute to prognosis and decision-making for treatments or lack of. Typically, such descriptions are only available via studies focused on one or two disorders in particular [14,15,18,22,23,24], whereas compilations of descriptions for small ruminants are less commonly incorporated within one paper [25].

In this study, reproductive disorders in females occurred in less than 7.00% of the animals (223/3394), with a greater occurrence in does than ewes (8.98% vs. 4.14%). In contrast, however, studies carried out in India and Iran reported greater absolute values of 23.3% (154/660) and 16.6% (108/648) for goats and sheep, respectively [26]. Perhaps crucially, such pathological conditions were described as gross genital lesions and observed at slaughterhouses, meaning that the greater frequency of cases may be related to the culling of adult females presenting low reproductive performance. Similarly, greater sensitivity is expected from a *post mortem* evaluation of reproductive pathologies when compared to ultrasound exams. 

Hydrometra stood out as significant among the reproductive disorders that developed in does, representing 50.98% (78/153) of those found and present in 4.58% (78/1704) of the total animals evaluated. This is a disorder that especially impairs the reproductive efficiency of dairy goat flocks and has also been reported in even greater frequencies [14,23,27]. Maia et al. [14] highlighted the rates of inbreeding that have occurred in the Saanen breed, which may have resulted in a genetic predisposition for hydrometra. In addition to the effects of breeding, other risk factors associated with hydrometra include aging; the use of hormonal protocols for estrus induction; the close presence of dogs and/or cats influencing embryo or fetal loss due to infection and, consequently, the development of hydrometra; and also having a mother with hydrometra [28,29,30]. Equally, follicular cysts and hydrosalpinx can also occur in association with hydrometra, meaning that reproductive efficiency can still become impaired after uterine emptying [14,31]. With this in mind, looking out for associated ovarian and uterine tube pathologies is important, together with performing a new ultrasound scan after hydrometra treatment, when the great uterine size prevents ovarian evaluation.

Recent fetal loss was among the main and medium findings in ewes (22.85%) and does (8.49%), respectively, which differs from slaughterhouse findings that showed endometritis and metritis to be the main factors for ewes [3]. This disorder is characterized by a fetus without heartbeats, according to an ultrasound scan [7,25], which can occur at any time throughout pregnancy, due to various causes related to both individual and flock-level factors. The non-infectious causes can include advanced age, multiple pregnancies, food restriction during pregnancy, mineral deficiencies, heat stress, inadequate handling, consumption of toxic drugs or plants, and also genetic influences [32,33,34,35]. In addition, infectious diseases such as toxoplasmosis, leptospirosis, and chlamydiasis are also associated with the occurrence of fetal mortality in goats and sheep [24,36]. Ultrasound, therefore, can contribute to the diagnosis of embryonic and fetal losses which, when occurring at levels above the expected flock frequency, can result in an early clinical investigation and intervention that may reduce productivity losses.

Cystic endometrial hyperplasia or glandular endometrial cysts are consequences of hormonal disorders, especially hyperestrogenism, which are rare in ruminants and more common in cats and dogs [37,38]. This pathology differs from the endometrial cysts, which are primarily found in senile mares, as a result of lymphatic obstruction associated with fibrosis and dilatation [37,39]. In ruminants, these complications are usually related to the constant and prolonged intake of phytoestrogens, which are present in poisonous plants and can also be produced by fungi [40,41]. In this respect, if there is a high occurrence of this pathology within a flock then it is important to look for specific plant genders (e.g., Trifolium and Medicago), or the effects of estrogenic fungi present in pasture or silage. When isolated cases are found, such as those in the present study (5/397 ewes in flock A; 1/33 in flock C; and 8/474 in flock G; for goats 1/98 in flock R and 4/397 in flock S), the causes may be associated with other conditions, such as uterine tumors or cystic ovarian degeneration [37], as well as being a result of individual sensibility to phytoestrogens. Finally, when identified in its initial stages, due to the presence of scarce small cysts, any complications are unlikely to inhibit the pregnancy, meaning that avoiding the phytoestrogens is the crucial factor for interrupting progression. The more numerous and larger the cysts, however, the more damaged the tissue, with alterations in the glands, stroma, and intercaruncular areas responsible for compromising the pregnancy [38].

By using the US assessment, it is possible to characterize testicular enlargement, parenchyma loss, the presence of masses and cysts, and modifications in echogenicity and echotexture, as well as the presence of intratesticular and extra testicular lesions [8,25]. Among these, testicular microlithiasis is a common finding in goat buck and ram testicles [8,22]. This development is usually related to aging factors, which do not compromise sperm quality; unlike in humans, where it may be related to neoplasia [42]. Equally, it is important to highlight that testicular microlithiasis only consists of hyperechogenic dots within the normal parenchyma; however, when an acoustic shadow is formed by this structure, a degenerative calcification is likely to blame [15], which may compromise sperm production. An US scan, therefore, allows for an early diagnosis of testicular lesions and prognosis prediction.

Additionally, our current findings can be utilized to guide further studies focused on the prevention and control of various reproductive disorders, as well as predisposing factors.

## 5. Conclusions

Reproductive disorders occurred in less than 7% of the female animals studied. Does presented more reproductive disorders than ewes, with hydrometra as the main finding. In ewes, recent fetal loss and cystic endometrial hyperplasia were identified as the main factors. In goat bucks and rams, testicular microlithiasis was the main finding. Finally, this paper presents a description of the most common ultrasonographic findings for small ruminants, serving as a guide for practitioners performing the diagnosis of such disorders, and ideally leading to an improvement of prognosis and, consequently, choosing the most effective treatment.

## Data Availability

Not applicable.

## Supplementary Materials

The following supporting information can be downloaded at: https://www.mdpi.com/article/10.3390/vetsci9110599/s1, Video S1: follicular cyst; Video S2: luteal cyst; Video S3: ovarian hypoplasia; Video S4: ovarian tumors; Video S5: paraovarian cyst; Video S6: hydrosalpinx; Video S7: salpingitis and endometritis; Video S8: cystic endometrial hyperplasia; Video S9: endometritis; Video S10: metritis; Video S11: hydrometra; Video S12: pyometra; Video S13: uterine torsion; Video S14: uterine tumors; Video S15: cervicitis; Video S16: paracervical abscess; Video S17: embryonic or fetal loss (aseptic form); Video S18: embryonic or fetal loss (septic form); Video S19: fetal maceration; Video S20: retained placenta; Video S21: recent embryonic fetal loss; Video S22: hydrocele; Video S23: testicular degeneration; Video S24: testicular microlithiasis; Video S25: testicular tumors; and Video S26: varicocele.

## References

[1] Scott P.R. (2012). Applications of diagnostic ultrasonography in small ruminant reproductive management. Anim. Reprod. Sci..

[2] Stieger-Vanegas S.M., McKenzie E. (2021). Imaging of the Urinary and Reproductive Tract in Small Ruminants. Vet. Clin. North Am. Food Anim..

[3] Khodakaram-Tafti A., Davari A. (2013). Congenital and acquired abnormalities of reproductive tract of non-pregnant ewes slaughtered in Fars province, Iran. Iran. J. Vet. Res..

[4] Ladds P.W. (1993). Congenital abnormalities of the genitalia of cattle, sheep, goats, and pigs. Vet. Clin. N. Am. Food Anim..

[5] Palmieri C., Schiavi E., Salda L.D. (2011). Congenital and acquired pathology of ovary and tubular genital organs in ewes: A review. Theriogenology.

[6] Bartlewski P.M. (2019). Applications of Doppler ultrasonography in reproductive health and physiology of small ruminants. Rev. Bras. Reprod. Anim..

[7] Gonzalez-Bulnes A., Pallares P., Vazquez M.I. (2010). Ultrasonographic Imaging in Small Ruminant Reproduction. Reprod. Domest. Anim..

[8] Gouletsou P.G. (2017). Ultrasonographic examination of the scrotal contents in rams. Small Rumin. Res..

[9] Crilly J.P., Politis A.P., Hamer K. (2017). Use of ultrasonographic examination in sheep veterinary practice. Small Rumin. Res..

[10] Aziz D.M., Lazim E.H. (2012). Transabdominal ultrasonography in standing position for pregnancy diagnosis in Awassi ewes. Small Rumin. Res..

[11] Barbagianni M.S., Ioannidi K.I., Vasileiou N.G.C., Mavrogianni V.S., Orfanou D.C., Fthenakis G.C., Valasi I. (2017). Ultrasonographic examination of pregnant ewes: From early diagnosis of pregnancy to early prediction of dystocia. Small Rumin. Res..

[12] Erdogan G. (2012). Ultrasonic assessment during pregnancy in goats—A review. Reprod. Domest. Anim..

[13] Ioannidi K.S., Mavrogianni V.S., Valasi I., Barbagianni M.S., Vasileiou N.G.C., Amiridis G.S., Orfanou D.C. (2017). Ultrasonographic examination of the uterus of ewes during the post-partum period. Small Rumin. Res..

[14] Maia A.L.R.S., Brandão F.Z., Souza-Fabjan J.M.G., Veiga M.O., Balaro M.F.A., Facó O., Fonseca J. (2018). Transrectal ultrasound evaluation in tropical dairy goats: An indispensable tool for the diagnosis of reproductive disorders. Trop. Anim. Health Prod..

[15] Cosentino I.O., Balaro M.F.A., Carvalho A.B.S., Trevizan J.T., Brandão F.Z., Fava C.D. (2019). Metastatic seminoma in a Male alpine goat: Clinical and histopathological approach. Acta Sci. Vet..

[16] Elbaz H.T., Razek E.M.A. (2019). Ultrasonographic Measurements of Reproductive Organs of Male Goat during Non- breeding Season. PSM Vet. Res..

[17] Elbaz H.T., Razek E.M.A. (2019). Ultrasonographic monitoring of reproductive organs of barki rams during early non-breeding season. J. Adv. Vet. Res..

[18] Espírito Santo C.G., Balaro M.F.A., Santos J.D.R., Correia L.F.L., Souza C.V., Taira A.R., Costa M.M.C.P., Carvalho A.B.S., Ungerfeld R., Brandão F.Z. (2020). Semen quality, testosterone values, and testicular and accessory gland parameters in rams receiving sustained stimulation with low doses of buserelin. Anim. Prod. Sci..

[19] Archana S., Vijay M., Anita B., Indu V. (2013). Pathological study on occurrence of various reproductive diseases in goats at Rajasthan. J. Immunol. Immunopathol..

[20] Durrani A.Z., Kamal N. (2009). Prevalence of genital tract problems in clinical cases of various species of animals. J. Anim. Plant. Sci..

[21] Sultan A., Islam M.R., Yadav R.K., Akhter R., Ahmed J.U. (2015). Prevalence of different reproductive disorders of small ruminants in five upazillas of Mymensingh district. Asian J. Med. Biol. Res..

[22] Cardilli D.J., Toniollo G.H., Pastore A.A., Canola J.C., Mercadante M.E.Z., Oliveira J.A. (2009). Padrão Ultrassonográfico Do Parênquima, Mediastino E Túnicas Testiculares Em Bovinos Jovens Da Raça Nelore. Cienc. Anim. Bras..

[23] Maia A.L.R.S., Brandão F.Z., Souza-Fabjan J.M.G., Veiga M.O., Balaro M.F.A., Siqueira L.G.B., Fonseca J.F. (2018). Hydrometra in dairy goats: Ultrasonic variables and therapeutic protocols evaluated during the reproductive season. Anim. Reprod. Sci..

[24] Ridler A.L., Vallee E., Corner R.A., Kenyon P.R., Heuer C. (2015). Factors associated with fetal losses in ewe lambs on a New Zealand sheep farm. N. Z. Vet. J..

[25] Balaro M.F.A., Maia A., Oliveira M.E.F., Cajueiro J.F.P., Andrade A.B.P., Brandão F.Z. (2019). Diagnóstico ultrassonográfico de distúrbios reprodutivos em pequenos ruminantes. Rev. Bras. Reprod. Anim..

[26] Beena V., Pawaiya R.V.S., Shivasharanappa N., Gururaj K., Gupta V.K., Gangwar N.K., Singh R. (2015). Occurrence of pathological conditions in the female genitalia of goats. Indian J. Vet. Pathol..

[27] Almubarak A.M., Abass N.A.E., Badawi M.E., Ibrahim M.T., Elfadil A.A., Abdelghafar R.M. (2018). Pseudopregnancy in goats: Sonographic prevalence and associated risk factors in Khartoum State, Sudan. Vet. World.

[28] Hesselink J.W., Elving L. (1996). Pedigree analysis in a herd of dairy goats with respect to the incidence of hydrometra. Vet. Q..

[29] Maia A.L.R.S., Silva M.R., Brandão F.Z., Souza-Fabjan J.M.G., Faria L.S., Côrtes L.R., Fonseca J.F. (2019). Epidemiological survey and risk factors associated with hydrometra in dairy goat herds. Small Rumin. Res..

[30] Wittek T., Erices J., Elze K. (1998). Histology of the endometrium, clinical-chemical parameters of the uterine fluid and blood plasma concentrations of progesterone, estradiol-17β and prolactin during hydrometra in goats. Small Rumin. Res..

[31] Souza J.M.G., Maia A.L.R.S., Brandão F.Z., Vilela C.G., Oba E., Bruschi J.H., Fonseca J.F. (2013). Hormonal treatment of dairy goats affected by hydrometra associated or not with ovarian follicular cyst. Small Rumin. Res..

[32] Czopowicz M., Kaba J., Szaluś-Jordanow O., Nowicki M., Witkowski L., Frymus T. (2012). Multivariate model for the assessment of risk of fetal loss in goat herds. Pol. J. Vet. Sci..

[33] Engeland I.V., Waldeland H., Andresen Ø., Løken T., Björkman C., Bjerkås I. (1998). Foetal loss in dairy goats: An epidemiological study in 22 herds. Small Rumin. Res..

[34] Engeland I.V., Waldeland H., Andresen Ø., Tverdal A. (1997). Foetal loss in dairy goats: An epidemiological study in 515 individual goats. Anim. Reprod. Sci..

[35] Holler L.D. (2012). Ruminant Abortion Diagnostics. Vet. Clin. North Am. Food Anim..

[36] Buxton D., Henderson D. (1999). Infectious abortion in sheep. Practice.

[37] Fatima B. (2011). Cystic Endometrial Hyperplasia in Algerian Goats and Ewes. Vet. Scan.

[38] Rizzoli D.J., Moran A.R. (1977). Permanent clover infertility in ewes. Aust. Vet. J..

[39] Radi Z.A. (2005). Endometritis and cystic endometrial hyperplasia in a goat. J. Vet. Diagn. Investig..

[40] Riet-Correa F., Pfister J., Schild A., Wierenga T. (2011). Poisoning by Plants, Mycotoxins, and Related Toxins.

[41] Wocławek-Potocka I., Mannelli C., Boruszewska D., Kowalczyk-Zieba I., Waśniewski T., Skarżyński D.J. (2013). Diverse Effects of Phytoestrogens on the Reproductive Performance: Cow as a Model. Int. J. Endocrinol..

[42] Leblanc L., Lagrange F., Lecoanet P., Marçon B., Eschwege P., Hubert J. (2018). Testicular microlithiasis and testicular tumor: A review of the literature. Basic Clin. Androl..

